# *Biomphalaria pfeifferi* Snails and Intestinal Schistosomiasis, Lake Malawi, Africa, 2017–2018

**DOI:** 10.3201/eid2503.181601

**Published:** 2019-03

**Authors:** Mohammad H. Alharbi, Charlotte Condemine, Rosie Christiansen, E. James LaCourse, Peter Makaula, Michelle C. Stanton, Lazarus Juziwelo, Seke Kayuni, J. Russell Stothard

**Affiliations:** Ministry of Health, Qassim, Saudi Arabia (M.H. Alharbi);; Liverpool School of Tropical Medicine, Liverpool, UK (M.H. Alharbi, C. Condemine, R. Christiansen, E.J. LaCourse, S. Kayuni, J.R. Stothard);; Research for Health Environment and Development, Mangochi, Malawi (P. Makaula);; Lancaster University Medical School, Lancaster, UK (M.C. Stanton);; Ministry of Health, Lilongwe, Malawi (L. Juziwelo);; Medical Aid Society of Malawi, Blantyre, Malawi (S. Kayuni)

**Keywords:** Epidemiology, Lake Malawi, Schistosoma mansoni, Kato-Katz, CCA dipsticks, Biomphalaria pfeifferi, snail, fluke, parasites, Africa

## Abstract

Two surveys conducted in 2017 and 2018 demonstrated *Biomphalaria pfeifferi* snails in Lake Malawi in Africa. Epidemiologic examination of 175 local children at 3 primary schools confirmed emergence of intestinal schistosomiasis. These findings highlight autochthonous transmission of *Schistosoma mansoni* flukes in Lake Malawi and the need to revise international travel advice.

Throughout sub-Saharan Africa, *Biomphalaria pfeifferi* snails are freshwater intermediate hosts for *Schistosoma mansoni* blood flukes, which cause intestinal schistosomiasis (*1*). Geographic distribution of *B. pfeifferi* snails delineates actual or potentially active zones of *S. mansoni* fluke transmission (*2*). Other than a report of a single *Biomphalaria* shell at Karonga in the far northern portion of Lake Malawi (*3*), considered to be from a marginal swamp (*4*), *B. pfeifferi* snails have not previously been found in Lake Malawi (*5*). However, in November 2017, during malacologic surveillance for intermediate hosts of schistosomiasis in the Mangochi District, Malawi, along the southernmost tip of Lake Malawi, 2 discrete populations of *Biomphalaria* snails were unexpectedly encountered in submerged beds of *Vallisneria* spp. plants (Figure, panel A). DNA sequence analysis of the mitochondrial cytochrome oxidase subunit 1 (*cox*1) (*6*) indicated that the *cox*1 sequences (1,006 bp) of those snails differed from sequences of *B. pfeifferi* snails from Chiweshe, Zimbabwe (GenBank accession nos. DQ084829 [HCO/LCO region] and DQ084872 [Asmit1/2 region]) by only 3 synonymous single-nucleotide polymorphisms.

**Figure:**
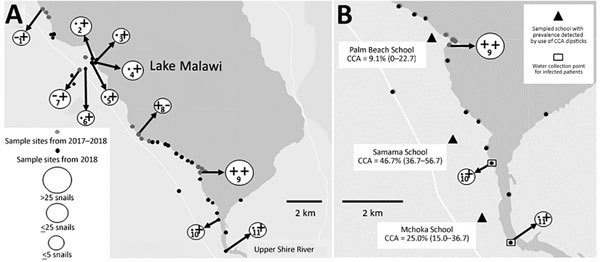
Locations sampled for *Biomphalaria pfeifferi* snails and of 3 primary schools where children were tested for intestinal schistosomiasis in the region of Lake Malawi, Africa. A) Locations sampled for *B. pfeifferi* snails in November 2017 (gray dots) and May 2018 (black dots), Lake Malawi, Africa. + indicates snails present, – indicates snails absent, and ● indicates site not sampled; symbol position indicates year of sampling (left, 2017; right, 2018). Numbers within circles indicate site numbers. Collected snail numbers are indicated by circle size. In 2017, snails were collected at 2 sites and not collected at 12 sites; in 2018, snails were collected at 10 sites and not collected at 47 sites. On each sampling occasion, >50 *B.* *pfeifferi* snails were collected at site 9. Coordinates of *B. pfeifferi*–positive sites: site 1, 14.27752°S, 35.10419°E; site 2, 14.31371°S, 35.14174°E; site 3, 14.31424°S, 35.14383°E; site 4, 14.31354°S, 35.14424°E; site 5, 14.31568°S, 35.14030°E; site 6, 14.32033°S, 35.13613°E; site 7, 14.32100°S, 35.13072°E; site 8, 14.36919°S, 35.17629°E; site 9, 14.39363°S, 35.22104°E; site 10, 14.42708°S, 35.23349°E; and site 11, 14.44928°S, 35.23890°E. B) Location of the 3 sampled primary schools (Palm Beach, 14.391346°S, 35.215137°E; Samama 14.417465°S, 35.217580°E; Mchoka 14.439481°S, 35.220644°E) showing local prevalence (% [95% CI]) of intestinal schistosomiasis indicated by *Schistosoma mansoni* circulating cathodic antigen (CCA) detected by urine dipstick. Water collection sites pinpointed by 2 *Schistosoma* egg–positive children from Samama and Mchoka Schools are indicated.

In May 2018, to confirm *B. pfeifferi* colonization within the lake and suspected risk for intestinal schistosomiasis, we undertook a conjoint malacologic and parasitologic survey with ethics approvals from the Liverpool School of Tropical Medicine, UK (application 17-018) and the Ministry of Health and Population, Malawi (application 1805). Reinspection of all prior malacologic sampling locations and another 43 sites found further populations of *B. pfeifferi* snails (Figure, panel A); large numbers (>50), along with innumerable dead shells, were again found at site 9. All snails were inspected for shedding cercariae, and although cercariae from snails at site 5 were seen, identification by microscopy (×100) was unsuccessful. Supplementary analysis indicated that *cox*1 sequences from 9 snails from sites 2, 5, 7, 10, and 11 were identical.

We conducted an epidemiologic survey of 175 school children, 5–15 years of age, equal numbers of boys and girls, from 3 primary schools closest to site 9 (Figure, panel B). Mean prevalence of intestinal schistosomiasis, calculated by detection of *S. mansoni* circulating cathodic antigen (CCA) on urine dipstick testing, was 34.3% (95% CI 27.9–41.3); prevalence rates by school were Samama, 46.7% (95% CI 36.7–56.7); Mchoka, 25.0% (95% CI 15.0–36.7); and Palm Beach, 9.1% (95% CI 0.0–22.7). We requested fecal samples from 60 *S. mansoni*–positive children and received samples from 46. Duplicate Kato-Katz examinations confirmed *S. mansoni* ova in 7 children; infection intensities were graded as light (<100 eggs/g feces). All urine samples were inspected for *S. haematobium* ova by syringe filtration (10 mL); general prevalence was 14.9% (95% CI 9.8–20.1); 52% of these samples were also positive by CCA urine dipstick, indicative of *S. mansoni* co-infection. To further determine autochthonous transmission of *S. mansoni* flukes*,* 2 egg-positive children from Samama and Mchoka took us, on foot, to the shoreline where they regularly swam, which corresponded to snail collection sites 10 and 11 (Figure, panel B). Children who were positive for either *S. mansoni* CCA or *S. haematobium* eggs received praziquantel (40 mg/kg). 

Colonization of *B. pfeifferi* snails in Lake Malawi and surrounding water is of concern, especially because active *S. mansoni* infections were found in local children. This finding highlights emergence of intestinal schistosomiasis, not previously documented here (*5*,*7*,*8*) or detected in this region by the most recent national survey (F. Fleming, Schistosomiasis Control Initiative, Imperial College London; 2017 Dec 20; pers. comm).

Intestinal schistosomiasis has been detected in children ≈150 km away, along the shoreline of the Lower Shire River (*9*). Finding snails and infected children in Mangochi District suggests recent ecologic and epidemiologic change. In May 2018, the lake was ≈75–80 cm higher than in November 2017, which perhaps favored detection of *B. pfeifferi* snails in the previously more accessible *Vallisneria* plant beds. Seasonal dynamics, such as lake level fluctuations, are well known, along with longer duration perturbations of the lake biota, either induced by climate change or mediated by anthropogenic activities. These changes have altered transmission of urogenital schistosomiasis (*10*); overfishing, particularly of the molluscivorous fish *Trematocranus placodon*, is changing the distribution of many freshwater snails (*5*).

Local aquaculture of fish (e.g., *Oreochromis* spp., called chambo) through use of water pumped inland from the lake has created novel, permanent water bodies colonized by *B. pfeifferi* snails (e.g., sites 2–7), which may now (re)seed snails into the lake for further establishment. Absence of *cox*1 genetic diversity in the *B. pfeifferi* snails we sampled implies a limited number or even a single founder event, but as conditions for autochthonous transmission became favorable, after introduction of *S. mansoni* flukes, intestinal schistosomiasis in local schoolchildren has emerged. This finding is of substantial public health concern in light of current control efforts, which consist only of annual praziquantel distribution in schools (*7*,*8*). We recommend increased surveillance of snails and characterization of schistosomes, along with intensified control interventions to arrest further spread of intestinal schistosomiasis. We also recommend revising and updating health and travel advice given to shoreline community residents and tourists who use the lake.
